# PM_2.5_ and Cardiovascular Diseases in the Elderly: An Overview

**DOI:** 10.3390/ijerph120708187

**Published:** 2015-07-16

**Authors:** Chenchen Wang, Yifan Tu, Zongliang Yu, Rongzhu Lu

**Affiliations:** 1 Department of Clinical Laboratory Medicine, School of Medicine, Jiangsu University, 301 Xuefu Road, Zhenjiang 212013, China; E-Mails: 2434009969@qq.com (C.W.); 501875090@qq.com (Y.T.); 2 Department of Cardiology, The First People’s Hospital of Kunshan Affiliated to Jiangsu University, 91 Qianjin West Road, Kunshan 215132, China; 3 Department of Preventive Medicine and Public Health Laboratory Sciences, School of Medicine, Jiangsu University, 301 Xuefu Road, Zhenjiang 212013, China; 4 Center of Experimental Research, The First People’s Hospital of Kunshan Affiliated to Jiangsu University, 91 Qianjin West Road, Kunshan 215132, China

**Keywords:** PM_2.5_, Cardiovascular disease (CVD), the elderly, susceptibility

## Abstract

*Background*: Cardiovascular disease (CVD) is the leading cause of mortality and morbidity in the elderly and the ambient concentration of PM_2.5_ has been associated with several cardiovascular diseases. *Methods*: We describe the present state of planetary air pollution, analyze epidemiological studies linking PM_2.5_ and CVD, and discuss multiple pathophysiological mechanisms linking PM_2.5_ and CVD. *Results*: A few epidemiological studies show that the elderly appear specifically susceptible to adverse cardiovascular effects triggered by PM_2.5_ exposure. Plausible pathophysiological mechanisms include inflammatory dysfunction, oxidative stress, abnormal activation of the hemostatic system and disturbance of the autonomic nervous system. *Conclusions*: An in-depth knowledge of the chemical compounds, pathophysiological mechanisms, and epidemiological studies of PM_2.5_ are recommended to understand this important and modifiable factor contributing to geriatric CVD burden. We offer public health recommendations to reduce this preventable cause of disease and death.

## 1. Introduction

Cardiovascular diseases (CVD) include disorders of the heart (arrhythmias, coronary vessel and vascular disease, heart failure) and blood vessels (peripheral arterial diseases and venous thrombosis), particular those supplying the brain (ischemic and hemorrhagic stroke). Together, these disorders constitute the leading cause of death across the globe, with low- and middle-income countries most heavily affected. According to the World Health Organization (WHO), each year about 17.3 million people die of cardiovascular disease, accounting for 30% of all deaths [1]. In addition to genetic and behavioral risk factors (unhealthy diet, physical inactivity, tobacco, and alcohol use), the inhalation of air containing fine particulate matter (particle size less than or equal to 2.5 μm) is associated with CVD. Some researchers including Brook [2] have proven that PM_2.5_ is a modifiable exposure factor that contributes to cardiovascular morbidity and mortality. Elderly people have the highest rates of CVD and thus are the most susceptible population [3]. We discuss plausible PM_2.5_-related pathophysiological mechanisms of CVD and epidemiological studies linking PM_2.5_ and CVD, especially in susceptible people, to make recommendations for future public health and reduce this avoidable cause of disease and death.

## 2. Review of PM_2.5_

Atmospheric particulate matter (PM) is classified into four categories according to aerodynamic diameter: Total suspended particulate (TSP ≤ 100 μm); particulate matter (≤10 μm); fine particulate matter (≤2.5 μm), and ultrafine particles (≤0.1 μm). PM_2.5_ refers to atmospheric particles with diameters less than or equal to 2.5 micrometers. The influence of PM on humans depends on particle size, which is linked with its aerodynamic diameter (AD) (Table 1). The range of most PM_10_ particles is from 2.5 μm to 10 μm while PM_2.5_ and PM_0.1_ have ADs ≤ 2.5 μm and ≤0.1 μm. PM_2.5_ and PM_0.1_ particles may permeate the lung alveoli and enter into the bloodstream, and then cause adverse health effects [4,5,6]. Compared with PM_10_, PM_2.5_ has a small particle size, light quality and a relatively large specific surface area [7]. The smaller particle size may pose a higher risk for systemic cardiovascular effects [8].

**Table 1:** Influences of particle size on human health.

Particulate	Particle Size (≤μm)	Influences on Human Health
PM_100_	100	Persist in the air and no evidence of adverse effects on human health
PM_10_	10	Enter the respiratory system, deposit in the respiratory tract and cause respiratory diseases
PM_2.5_	2.5	Get into the alveoli through the respiratory tract and then enter into the blood circulation, causing various diseases.
PM_0.1_	0.1	

The major sources of particulate matter are broadly divided into two parts, human activities and natural phenomena, including wildfires, volcanoes, and land dust [9]. In addition, chemical reactions of primary emissions in the atmosphere cause the formation of secondary pollutants and the composition varies according to the pollution source [9,10,11].

**Figure 1:**
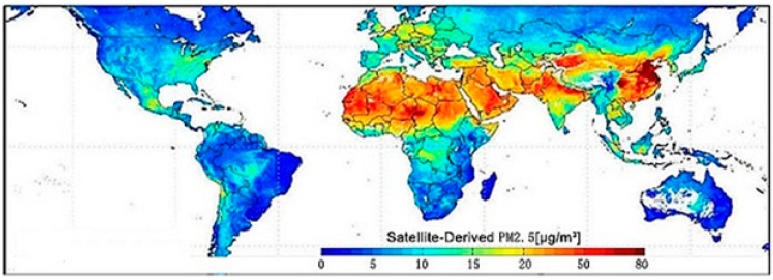
The global distribution of PM_2.5_ averaged over 2001–2006 (Credit: Dalhousie University, Aaron van Donkelaar, http://www.nasa.gov/topics/earth/features/healthappoing.html).

We may recall the Great Smog event in London in December 1952, when severe air pollution from domestic and industrial coal burning caused thousands of excess deaths, especially among the very young and elderly, and led to the passage in the UK of the 1956 Clean Air Act [12,13]. Nowadays, PM_2.5_ is considered the main culprit for the adverse effects of air pollution on human health [2]. PM_2.5_ pollution affects the whole planet, particularly densely populated metropolitan areas of eastern and southern China, northern India, and the emerging countries of South-East Asia. Parts of Europe and America are not spared as well (Figure 1). The concentration of PM_2.5_ in the area mentioned above may even exceed 100 μg/m^3^ [14]. By contrast, in 2012, the revised annual arithmetic mean in the USA was 12 μg/m^3^ for primary PM_2.5_ and 15 μg/m^3^ for secondary PM_2.5_, with a 24-hour 98th percentile value of 35 μg/m^3^ for both [15].

## 3. Plausible Pathophysiological Mechanisms Linking PM_2.5_ Exposure and CVD

Several studies suggest that the elderly are especially susceptible to the harmful effects of PM exposure [2]. Older adults also represent a potentially susceptible population compared with children or younger adults because of the higher prevalence of preexisting cardiovascular and respiratory diseases. Epidemiological studies assessing the relationship between air pollution and CVD have appeared in the past 20 years [16]. High concentrations of PM_2.5_ have been associated with morbidity and mortality in both short- and long-term epidemiological studies [17].

Air particulate matter enters the human body mainly in two ways: (1) PM_2.5_ enters the respiratory system causing pulmonary and systemic inflammation, oxidative stress, affecting the coagulation system, changing autonomic nerve function, injuring the vascular endothelium and affecting vasomotor function; (2) Part of the particulate matter causes the above reaction through other routes, such as entering into the circulatory system via the digestive tract. Animal studies have shown that PM_2.5_ can be devoured by macrophages and endothelial cells, indicating that air pollution may have direct effects on the vascular system [4,5,6,18].

The relationship between air pollution and respiratory diseases, such as asthma and chronic obstructive pulmonary disease, is well established. Nevertheless, the relationship between air pollution and CVD remains unclear. The plausible pathophysiological mechanisms can be divided into the following several aspects [19].

### 3.1. Oxidative Stress and Inflammation

Both animal and human studies [20,21,22,23,24] have shown that inhaled particles may cause inflammation of the respiratory tract; in particular, PM_2.5_ inhalation can lead to the occurrence of systemic inflammation, increasing the risk of cardiovascular stress. PM_2.5_ can enter the alveolar epithelium, cause local inflammation and oxidative stress, resulting in the release from lung cells into bronchial fluid and the blood stream of several inflammatory mediators, such as IL-6, IL-8, TNF-α and interferon-γ [25]. These spread to the general circulation where they can plausibly modulate systemic effects. When mice are exposed to PM_2.5_ for six hours, there is increased expression of inflammation-related genes such as mRNA of TNF-α, TNF-β and IL-6, IL-8. A recent study finds that IL-6 can make a rapid response to air pollution and increase the production of C-reactive protein (CRP) [26]. CRP is the most important protein in acute phase reactants (APR), and it is also a sensitive indicator of inflammation associated with the risk of cardiovascular disease. Blood CRP increases in proportion to the concentration of PM_2.5_; for every increase of 100 μg/m^3^, blood CRP increases 8.1 mg/L [27]. Interestingly, coarse PM has been suggested to directly trigger inflammation by binding to toll-like receptor (TLR) 2 and 4 [28]. Higher TLR2 methylation may confer susceptibility to adverse cardiac autonomic effects of PM_2.5_ exposure in older individuals [29].

### 3.2. Abnormal Activation of the Haemostatic System

Another probably detrimental effect of PM_2.5_ exposure is an abnormal activation of the hemostatic system. Epidemiological studies have associated PM_2.5_ inhalation with venous thrombosis and a shorter prothrombin plasma-clotting time. The diffuse pulmonary inflammation caused by PM_2.5_ may spread to the circulatory system, causing abnormal activation of the hemostatic system [30]. Animal models and *in vitro* cell studies show that PM_2.5_ increases fibrinogen and tissue factors [31], and fine particulate matter in blood vessels can directly activate platelets. Platelets play an important role in thrombus formation and can make the blood hypercoagulable states. PM_2.5_ exposure may increase the risk of acute thrombosis, as in myocardial infarction and ischemic stroke. At the same time, fibrinogen is also an important risk factor for stroke [32].

### 3.3. Disturbance of Autonomic Nervous System

PM particles disturb the autonomic nervous system (ANS) [33]. Under normal circumstances, the rhythmic activity of the heart is controlled by the activity of autorhythmic cells in the sinoatrial node, which is regulated by the vagus nerve. Acute exposure to PM_2.5_ can stimulate the ANS and increase the risk of arrhythmia and acute cardiovascular events, with serious impact on the elderly [34]. A number of studies in a recent meta-analysis support an inverse association between PM exposure and heart rate variability [35]. Heart rate variability (HRV) refers to the cyclical changes of sinus rhythm, and it is an important index of tonicity and sympathetic-parasympathetic balance. Some research finds that exposure of PM_2.5_ is linked with HRV change in the elderly. Compared with exposure to clean air for two hours, HRV drops 35.7% after inhalation of 21.2~80.3 μg/m³ PM_2.5_ [36]. Moreover, in a 48-hour moving average, PM_2.5_ was found to have a strong effect on the decrement in HRV [37]. Additionally, the effects of PM_2.5_ on subjects with hypertension were larger than on the subjects without hypertension [38]. The decrease of HRV reflects direct perturbation of the cardiac autonomic nervous system, and may serve as a prelude to heart disease.

### 3.4. Injury of Endothelial Cells

Because vascular endothelial injury is an important pathological basis of cardiovascular disease, the damage of vascular endothelial cells by PM_2.5_ is one possible CVD mechanism. Cardiovascular endothelial cells treated *in vitro* with PM_2.5_ for 24 hours display adhesion molecules and apoptosis [39]. Cell mortality rates increase with PM_2.5_, suggesting that PM_2.5_ may damage the vascular endothelium, thereby causing CVDs. Recently, some studies suggest that specific metals may be important components responsible for PM_2.5_-induced cardiovascular effects and that the reduced capacity of endothelial repair may play a critical role [40].

## 4. Epidemiological Studies Linking PM_2.5_ and CVD in the Elderly

Though several studies have shown exposure to PM_2.5_ can increase risk of CVD [2,19,41,42,43,44,45,46,47,48,49,50,51,52,53,54,55,56,57], few studies focused on the elderly, one of susceptible subpopulations, Kan *et al*. found that risk of CVD exposed to PM_10_ increased 0.26%, compared with people 5–44 years of age or 45–64 years of age[58,59]. Another Chinese studies in Beijing showed that ambient PM_2.5_ adversely affected cardiac autonomic function of the elderly people with heart diseases [38]. Dominici and colleagues analyzed acute effect of fine particulate air pollution on elderly people (age > 65 years) and found that the largest association between PM_10_ and congestive heart failure, a 0.72% increase in risk per 10 μg/m^3^ elevation in same-day PM_10_ concentration [49]. This is more than the results reported by Brook with an increasing of 0.18% in the general population [2]. However, annual average exposure to higher levels of black carbon (per 0.26 μg/m^3^ elevation), a marker of traffic-related PM, was associated with a 1.1% increase in carotid intima media thickness (CIMT) in a cohort of elderly men living in the Boston area [60]. Short-term PM exposure has been more strongly associated with cardiac mortality among older individuals [61]. In an elderly population (aged 65 and older) across Eastern USA, researchers found that both short-term and long-term exposure were significantly associated with risk of deep vein thrombosis (DVT) [62]. While the majority of studies and expert-consensus opinions consistently agree that PM_2.5_ can increase the risk of CVD, it is noteworthy that some studies had not find relationships between PM_2.5_ and risk of CVD [63,64,65,66].

## 5. Conclusions

From a point of pathophysiology, the elderly is a susceptible population to cardiovascular injury by PM_2.5_ and a few epidemiological studies showed more risk of CVD increased in the elderly exposed to the same levels of PM_2.5_. Due to increasing of PM_2.5_ level and aged populations, more attention should be paid to CVD risk and PM_2.5_ exposure in the elderly. In addition, the relationship between PM_2.5_ concentration and CVD risk appears not to have a “safe” threshold. The “alarm” threshold periodically claimed by regulatory agencies during certain seasons should therefore be considered advisory, and efforts should be made to keep level of pollutants as low as possible [2]. Thus, besides of reducing air pollution, avoidance of exposure to PM_2.5_ pollution and societal and personal measures to reduce other risk factors should be recommended for the elderly [67,68]:

- Walking or cycling in parks or country roads instead of busy streets during rush hours.
- Using more public transport rather than private cars and motorbikes.
- Avoiding outdoor exercise at times of high PM_2.5_ (especially those with cardiorespiratory disorders).
- Using an air purifier to optimize indoor air quality.
- Choosing appropriate respirators that fit snugly on the face if air pollution is severe.
- Reducing other known cardiovascular risk factors, such as smoking and alcohol.
- Warranting more aggressive use of primary and secondary preventive therapies, including antiplatelet agents, lipid-lowering agents, and treatments for hypertension or diabetes.
